# Allogeneic Hematopoietic Stem Cell Transplantation in Myelofibrosis: Evolving Indications, Conditioning Strategies, and Outcomes

**DOI:** 10.3390/hematolrep18050061

**Published:** 2026-08-28

**Authors:** Caterina Alati, Stefano Botti, Francesca Cogliandro, Martina Pitea, Matteo Pacilli, Gaetana Porto, Giorgia Policastro, Annalisa Sgarlata, Maria Caterina Mico, Barbara Loteta, Laura Giordano, Giulia Santoro, Jessyca Germano, Massimo Martino

**Affiliations:** 1 Hematology and Stem Cell Transplantation and Cellular Therapies Unit (CTMO), Department of Hemato-Oncology and Radiotherapy, Grande Ospedale Metropolitano Bianchi-Melacrino-Morelli, 89133 Reggio Calabria, Italy; caterina.alati@ospedalerc.it (C.A.); francesca.cogliandro@ospedalerc.it (F.C.); matteo.pacilli@ospedalerc.it (M.P.); porto.tania25@gmail.com (G.P.); giorgia.policastro@ospedalerc.it (G.P.); annalisa.sgarlata@ospedalerc.it (A.S.); mariacaterina.mico@ospedalerc.it (M.C.M.); barbara.loteta@ospedalerc.it (B.L.); giordanolau@gmail.com (L.G.); jessicagermano@libero.it (J.G.); dr.massimomartino@gmail.com (M.M.); 2 Hematology Unit, Oncology and Advanced Technology Department, Azienda USL-IRCCS di Reggio Emilia, Via Amendola 2, 42122 Reggio Emilia, Italy; stefano.botti@ausl.re.it; 3 Hematology, Stem Cell Transplantation, Fondazione Policlinico Universitario Campus Bio Medico, 00128 Rome, Italy; giulia.santoro@unicampus.it

**Keywords:** myelofibrosis, allogeneic hematopoietic cell transplantation, conditioning regimen, treosulfan, JAK inhibitors, MRD monitoring, GITMO, myeloproliferative neoplasms

## Abstract

**Background/Objectives**: Myelofibrosis (MF) is a clonal myeloproliferative neoplasm driven by dysregulated JAK-STAT signaling, characterized by progressive marrow fibrosis, splenomegaly, constitutional symptoms, and increased risk of leukemic transformation. Allogeneic hematopoietic stem cell transplantation (allo-HCT) remains the only potentially curative intervention. In Italy, allo-HCTs for myeloproliferative neoplasms (MPN, i.e., myelofibrosis, polycythaemia vera, and essential thrombocythaemia combined) increased by 163% from 2015 to 2025; MF-specific procedure counts were not separately available in this registry export, so this figure should not be read as MF-specific, with MPN accounting for 8% of all allogeneic procedures (n = 171) in 2025, showing a 29.5% year-on-year increase from 2024. Concurrently, the demographic profile shifted, with individuals aged 60 and over representing 38% of all recipients, surpassing other age groups for the first time in 2025. **Results**: This review synthesizes current evidence on transplant indications and timing, pre-transplant management, donor selection, and conditioning regimen optimization, emphasizing the emerging role of treosulfan-based and dual-alkylator platforms, including the thiotepa–treosulfan–fludarabine (TTF) regimen. Post-transplant molecular MRD monitoring and relapse management are also discussed. Three-year overall survival in myelofibrosis ranges from about 59% to 67%, depending on donor type, with outcomes improving due to better patient selection, optimized conditioning, and supportive care. **Conclusions**: Prospective randomized trials are urgently needed to validate optimal conditioning intensity, the role of novel JAK inhibitors in the peri-transplant period, and MRD-guided pre-emptive strategies.

## 1. Introduction

Myelofibrosis (MF) is a BCR-ABL1-negative myeloproliferative neoplasm (MPN) characterized by clonal expansion of a hematopoietic stem cell harboring driver mutations in JAK2 (approximately 55–60% of cases), CALR (20–25%), or MPL (5–8%), all converging on constitutive JAK-STAT pathway activation [1,2,3,4]. Marrow fibrosis, ineffective hematopoiesis, splenomegaly, and systemic inflammatory cytokine overproduction dominate the clinical picture [5,6]. The disease exists in primary (PMF) and secondary forms arising from post-polycythemia vera (post-PV MF) or post-essential thrombocythaemia (post-ET MF), the latter classified collectively as secondary myelofibrosis (SMF) and associated with distinct prognostic scoring systems [7].

Allogeneic Hematopoietic Stem Cell Transplantation (Allo-HCT) remains the only intervention capable of eradicating the malignant clone, resolving marrow fibrosis, and reversing the cytokine milieu that drives constitutional symptoms [8]. Nonetheless, HCT is associated with substantial non-relapse mortality (NRM), and appropriate patient selection and timing remain central challenges [9,10]. The field has been transformed over the past decade by the introduction of JAK inhibitors—particularly ruxolitinib—as pre-transplant cytoreduction [11], expanded donor platforms including haploidentical transplantation with post-transplant cyclophosphamide (PTCy) [12], and refined conditioning strategies [10]. Most recently, treosulfan-based regimens have emerged as a promising alternative to busulfan-based protocols, with a potentially more favorable toxicity profile and comparable anti-disease efficacy [13].

The Italian Gruppo Italiano Trapianto di Midollo Osseo (GITMO) registry offers a comprehensive national perspective on trends in allo-HCT for MPN [14]. From 2015 to 2025, the absolute number of allogeneic procedures for the combined MPN category (myelofibrosis, polycythaemia vera, and essential thrombocythaemia) increased by 163%, rising from 65 to 171 per year, which represents the largest proportional growth among all indications during this period. In 2025, MPN accounted for 8% of the 2152 allogeneic transplants performed in Italy, a 29.5% increase from 2024 [15]. Alongside this growth, a significant demographic shift has occurred: the proportion of recipients aged 60 years or older increased from 14% in 2015 to 38% in 2025, and those aged 70 years or older increased more than tenfold (from 9 to 104 patients).

This review critically examines the evolving landscape of allo-HCT in MF, with particular emphasis on Italian and European data, recent registry analyses, and emerging therapeutic strategies.

## 2. Epidemiology of Allo-HCT in MPN: Italian and International Trends

GITMO registry data, exported on 13 April 2026 and spanning >50,000 cumulative allogeneic procedures since 1991 (n = 50,234), demonstrate sustained growth in overall transplant activity reaching 2152 procedures in 2025—a 3.5% increase over 2024 and 7.3% over 2023 [14]. Against this backdrop, the growth of MPN-directed transplantation is particularly striking.

As shown in Table 1, MPN transplant numbers have grown from 65 in 2015 to 171 in 2025, a 163% increase, contrasting sharply with stable or declining volumes in AML (−2.0%), ALL (−1.3%), and NHL/HD (−0.5%) over the same single-year interval (2024–2025). As this MPN category combines myelofibrosis, polycythaemia vera, and essential thrombocythaemia, and MF-specific case counts were not separately exportable from the registry, the 163% figure should be interpreted as an indication of overall MPN transplant growth rather than an MF-specific estimate; the data in Table 1 were exported directly from the GITMO registry rather than taken from a previously published report, and reflect hematological-malignancy indications only (severe aplastic anemia, for example, is not represented). The MDS/MPN category similarly grew by +15.8% year-on-year. This reflects not only disease-specific epidemiological trends, but also expanding referral criteria, improved pre-transplant optimization with JAK inhibitors, and broadening age thresholds. The demographic shift in the Italian transplant population is equally noteworthy. In 2025, recipients aged >60 years accounted for 38% of all procedures (n = 775), surpassing—for the first time in the GITMO registry—all other age groups, including the historically dominant 41–60 cohort (n = 757, 37%) [14]. Patients aged ≥70 years increased from 9 (1%) in 2015 to 104 (5%) in 2025, a 1056% increase in absolute numbers over a decade. This shift parallels international data: German registry data reported by Gagelmann and Kröger show a sustained rise in HCT for MDS and MPN with approximately 780 procedures in 2023 [8], and the US CIBMTR report by Spellman et al. documents progressive improvement in 3-year survival probability from 45.3% (2002–2006) to 62.1% (2017–2022) among 144,996 patients (*p* < 0.0001) [16].

## 3. Disease Biology and Risk Classification

### 3.1. Driver and High-Molecular-Risk Mutations

Driver mutations in JAK2 V617F, CALR (types 1 and 2), and MPL W515L/K are present in approximately 90% of MF patients and all promote dysregulated JAK-STAT signaling and cytokine overproduction [1,2,3,4]. Beyond driver mutations, high-molecular-risk (HMR) mutations—defined as mutations in ASXL1 (most common), EZH2, IDH1/IDH2, SRSF2, and U2AF1 Q157—are associated with significantly reduced overall survival and an increased risk of leukemic transformation [15,17,18,19,20,21]. TP53 biallelic mutations (multihit) define a very-high-risk subset in whom HCT should be considered regardless of clinical prognostic score.

### 3.2. Prognostic Scoring Systems

Multiple prognostic systems are used in routine clinical practice and transplant decision-making. The Dynamic International Prognostic Scoring System (DIPSS) and DIPSS-plus incorporate clinical variables and cytogenetic risk [22]. MIPSS70 and MIPSS70+ version 2.0 integrate HMR mutations and cytogenetic data for primary MF [23,24,25]; MYSEC-PM applies specifically to secondary MF with four risk categories [26,27]. The Myelofibrosis Transplant Scoring System (MTSS) is the sole transplant-specific score that incorporates donor source and Karnofsky performance status [28]. These tools are complementary: DIPSS/MIPSS70 guide the indication for HCT, while MTSS refines timing and donor selection.

## 4. Transplant Indications, Timing, and Patient Selection

### 4.1. Risk-Stratified Approach

The decision to proceed to allo-HCT and its timing depend critically on the balance between disease-related mortality risk and transplant-related morbidity and NRM. Current evidence, originally modelled by Cipkar et al. and synthesized by Gagelmann and Kröger, supports a risk-stratified approach [8,29]:

- DIPSS High Risk: immediate HCT is recommended; peak survival benefit at approximately 10 months from diagnosis;
- DIPSS Intermediate-2: immediate HCT; peak benefit at approximately 17 months;
- DIPSS Intermediate-1: individual evaluation; benefit window at approximately 21 months; MTSS score should guide decision;
- DIPSS Low Risk: non-transplant approach preferred; HCT benefit only apparent after 29–45 months of conservative management.

For SMF (post-PV or post-ET), MYSEC-PM intermediate-2 or high-risk disease constitutes a standard indication; MYSEC-PM intermediate-1 warrants individual assessment incorporating MTSS.

### 4.2. Molecular Risk and JAK Inhibitor Response

The presence of HMR mutations, particularly ASXL1 and TP53 multihit, can upgrade the HCT indication even in patients with low DIPSS scores [17,30]. A specific subgroup not otherwise addressed here is “triple-negative” MF (JAK2/CALR/MPL wild-type), which represents around 10% of cases; whether this molecular subtype independently informs risk stratification and the HCT decision remains debated, with differing conclusions reported in the literature [31,32]. The RR6 model evaluates response to ruxolitinib at 6 months: poor responders—defined by insufficient spleen volume reduction combined with transfusion dependence—should be promptly referred for HCT evaluation, as continued JAK inhibitor therapy confers diminishing benefit and may delay curative treatment [33].

### 4.3. Age Considerations

Advanced age is no longer considered an absolute contraindication to allo-HCT in MF (Table 2). CIBMTR data indicate a 3-year overall survival of approximately 55% in patients aged 70 years or older who undergo transplantation with good performance status and an HLA-matched donor, supporting individualized assessment rather than age-based exclusion [16]. The marked increase in elderly transplant recipients in the GITMO registry (from 1% to 5% aged ≥70 years over the decade) further illustrates this paradigm shift [14].

## 5. Pre-Transplant Management

### 5.1. JAK Inhibitor Therapy

The primary goal of pre-transplant therapy is to reduce disease burden—particularly splenomegaly and constitutional symptoms—to optimize fitness for conditioning and engraftment [34,35,36,37]. Ruxolitinib remains the cornerstone, with approximately 3 months of therapy generally recommended before HCT [38]. Ruxolitinib must be tapered rather than abruptly discontinued before conditioning to avoid the ruxolitinib discontinuation syndrome, a potentially severe inflammatory rebound characterized by fever, hypotension, and cytokine storm. Sustained splenic response correlates with improved post-transplant outcomes [39]. For thrombocytopenic patients (platelets < 100 × 10^9^/L), pacritinib or fedratinib is an appropriate alternative [40,41].

Momelotinib, recently approved for anemia-related symptoms, may reduce transfusion dependence in the pre-transplant setting. This use is extrapolated from momelotinib’s demonstrated effects on anemia and splenomegaly; the pivotal MOMENTUM trial was not designed to, and did not, provide direct evidence of a benefit on transplantation-specific endpoints, and its scope should be understood accordingly [42]. Non-responders should be assessed for second-generation JAK inhibitors or early HCT referral [43].

### 5.2. Splenomegaly Management

Bulky splenomegaly—generally defined as spleen length > 22 cm or volume > 3000 mL—is a significant risk factor for primary graft failure due to sequestration of progenitor cells [44]. Splenic irradiation (3–4 Gy in 2–3 fractions) has demonstrated significantly reduced relapse incidence compared with splenectomy in JAK inhibitor-refractory patients and can be integrated into the HCT algorithm or conditioning pathway [44,45]. Splenectomy remains complex, particularly for very large spleens, and is consistently associated with higher relapse risk in retrospective analyses [44].

## 6. Donor Selection

The choice of donor is critical and influences early post-transplant morbidity and long-term outcome [46]. Key principles from current evidence are summarised in Table 3.

The HLA-matched sibling donor (MSD) remains the preferred option, offering superior overall survival in the first 3 months post-HCT and lower early non-relapse mortality. Where the available sibling donor is older (e.g., >60 years), a younger 10/10 matched unrelated donor (MUD) may represent a preferable alternative in some cases; however, this should be regarded as an evidence-based consideration rather than an absolute rule, and donor comorbidities, stem-cell source, and center experience should also be taken into account. The use of haploidentical transplantation with post-transplant cyclophosphamide (PTCy) has increased substantially, from 3% of procedures in 2013 to 19% in 2019, according to CIBMTR registry data [47] (this specific trend is reported in the CIBMTR registry rather than in GITMO data). Reported 3-year overall survival with haploidentical HCT has varied across cohorts: a relatively small multicenter series (n = 69) reported approximately 72% [48], whereas larger contemporary registry analyses report lower figures of approximately 56–59% (CIBMTR, n = 566: 58.7%; an adjusted EBMT analysis additionally reports an HR of 1.42 for overall survival with haploidentical versus matched sibling donors) [49]. These differences likely reflect variation in patient selection, transplant era, conditioning intensity, GVHD prophylaxis, and registry-based versus single/multicenter study design; given its smaller sample size, the 72% figure should be interpreted cautiously and larger series may be more representative. Haploidentical HCT with PTCy nonetheless remains a viable alternative when a matched donor is unavailable, with several studies suggesting broadly comparable outcomes to MUD [48,49]. Cord blood transplantation is not recommended for MF due to the high risk of graft failure in this setting [8,46].

## 7. Conditioning Regimen Selection

### 7.1. Principles and Intensity

Conditioning regimens in MF must achieve three objectives: sufficient myeloablation to overcome the fibrotic marrow and enable donor engraftment; adequate immune suppression to prevent graft rejection; and anti-disease cytoreduction to minimize relapse risk [50]. The optimal balance between regimen intensity and NRM remains a central clinical challenge, particularly given the older and more comorbid profile of MF patients.

Registry data show broadly comparable overall survival between reduced-intensity conditioning (RIC) and myeloablative conditioning (MAC) regimens in MF. Contrary to earlier assumptions, the two largest comparative analyses to date found no significant difference in non-relapse mortality (NRM) between RIC and MAC, and only a non-significant trend toward higher relapse with RIC [51,52]. The single significant intensity-related benefit identified so far is graft-versus-host-disease-free, relapse-free survival (GRFS), which favoured MAC in the EBMT cohort (32.4% vs. 26.1%, *p* = 0.001); relapse itself was only a non-significant trend (*p* = 0.08) [51]. Fludarabine–busulfan remains the most commonly used backbone in both intensity strata.

### 7.2. The Role of Dual Alkylation and Chimerism Optimization

A unique feature of MF transplantation is the association between early full-donor chimerism and long-term disease control. Mixed chimerism at Day +30 post-HCT is associated with higher relapse incidence on univariate analysis compared with full-donor chimerism (40% vs. 14% cumulative relapse incidence, *p* = 0.01) [53]; however, in the multivariable model of the same study this variable did not retain statistical significance, so it should not be described as an independent predictor of relapse. The thiotepa–busulfan–fludarabine (TBF) regimen, utilizing dual alkylation, achieves full-donor chimerism in 87–93% of patients at Days +30, +60, and +90, compared with only 45% with single-alkylator conditioning (*p* < 0.0001), with a corresponding reduction in relapse from 43% (ONE-ALK) to 9% (TBF, *p* < 0.00001) and an improvement in 5-year disease-free survival from 43% to 63% (*p* = 0.01) [53].

### 7.3. Treosulfan-Based Conditioning: Emerging Evidence

Treosulfan, an alkylating prodrug structurally related to busulfan but non-enzymatically activated to epoxy derivatives at physiological pH, has been increasingly adopted in Europe [54]. Comparative clinical cohort data in acute myeloid leukemia and other myeloid malignancies (rather than preclinical models) demonstrate antileukemic activity equal to or greater than that of busulfan, with potentially more profound lymphodepletion, a more favorable cytokine milieu, and, consequently, less graft-versus-host disease [13]. Unlike busulfan, treosulfan has limited extramedullary toxicity [55]. Early single-center experience with treosulfan-based reduced-toxicity conditioning also demonstrated feasibility and encouraging survival outcomes in patients with myelofibrosis undergoing allo-HCT, providing the basis for subsequent registry-based and multicenter investigations [56]. Multiple registry and multicentre studies have evaluated treosulfan in MF. The EBMT Registry study by Robin et al. evaluated n = 530 myelofibrosis patients [57]. The study shows that treosulfan-based conditioning offers superior PFS and OS alongside lower NRM compared with high-dose busulfan, while maintaining similar relapse rates. The German registry study by Gagelmann et al. analyzed 1115 myelofibrosis patients undergoing HSCT to compare busulfan and treosulfan conditioning [58]: 4-year OS was 69% with RIC-treosulfan, with a similar relapse risk to busulfan-based regimens. High-dose treosulfan (≥42 g/m^2^) was independently associated with increased all-cause mortality (HR 1.61, *p* = 0.006); however, this hazard ratio refers to overall death, not to NRM specifically. In the same study, the NRM hazard for high-dose treosulfan was 1.48 (95% CI 0.98–2.23, *p* = 0.06), a non-significant result, indicating that the effect of dose intensity on NRM alone remains unproven and that dose optimization above 42 g/m^2^ should be interpreted cautiously. A recent systematic review by Nasiri et al. conflated this overall-mortality hazard with NRM and should not be relied upon for this specific comparison [59]; nonetheless, taken together the available registry and single-center data suggest that treosulfan-based conditioning may represent an effective and reasonably well-tolerated backbone for HSCT, though this conclusion is based mainly on retrospective evidence and requires prospective confirmation [59] (Table 4). 

### 7.4. The TTF Regimen: Rationale and Unresolved Questions

The thiotepa–treosulfan–fludarabine (TTF) double-alkylator regimen is a novel strategy that combines the immune-suppressive and myelodepleting effects of thiotepa with the reduced extramedullary toxicity of treosulfan and the immunosuppressive potency of fludarabine [63,64]. The proposed advantages include: (i) dual alkylation to enhance full-donor chimerism; (ii) disease control comparable to myeloablative conditioning [17]; (iii) the ability to modulate doses of treosulfan (30–42 g/m^2^) and thiotepa (5–10 mg/kg) according to patient fitness, facilitating use across a broad age and comorbidity spectrum; and (iv) substitution of busulfan, which is associated with hepatic veno-occlusive disease risk, with treosulfan to potentially reduce sinusoidal injury. However, critical questions remain unanswered in MF-specific populations. No prospective randomized trial has evaluated TTF versus standard FluBu2 or TBF in myelofibrosis. The optimal dose thresholds for treosulfan and thiotepa in this indication, the interaction with haploidentical donors, and the role of PTCy-based GVHD prophylaxis in the TTF platform require formal prospective investigation.

## 8. Post-Transplant Outcomes

**Figure 1:**
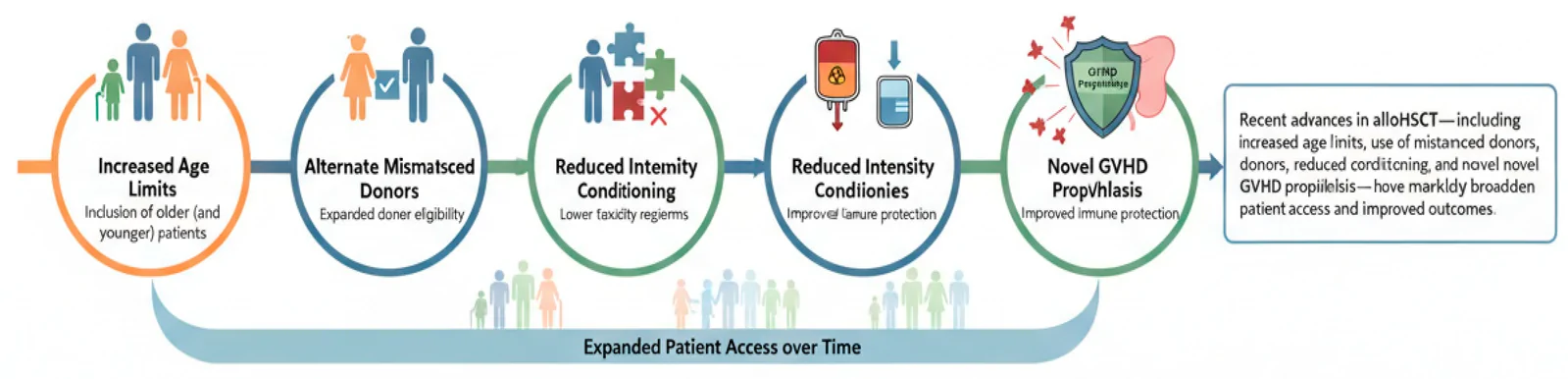
Evolution of allogeneic hematopoietic stem cell transplantation.

Current transplant outcomes in MF have improved considerably over the past two decades, reflecting advances in patient selection, conditioning optimization, CMV prophylaxis, and supportive care [8,16]. Key contemporary benchmarks are summarised in Table 5 and Figure 1.

CIBMTR data from 2017–2022 show 3-year OS of 67.4% for MF patients with matched related donors, 59.1% with matched unrelated donors, 58.7% with haploidentical donors, and 54.5% with mismatched unrelated donors [16]. These results compare favorably with those of other MPN subtypes in the haploidentical and mismatched settings (MF consistently outperforms them). Outcomes continue to improve: US data demonstrate a linear progression from 45.3% (2002–2006) to 62.1% (2017–2022) 3-year OS across 144,996 first allogeneic HCT recipients (*p* < 0.0001).

## 9. Molecular MRD Monitoring After HCT

Post-transplant molecular monitoring has become a cornerstone of MF management, outperforming traditional chimerism analysis as a predictor of relapse and long-term outcome. Complete clearance of driver mutations—particularly JAK2 V617F—at Day +30 post-HCT is the most powerful predictor of improved relapse incidence, disease-free survival, and overall survival [65,66,67].

### Recommended Monitoring Protocol

- Timepoints: Month 1, then every 3 months up to 1 year, then annually for detection of late relapse;
- Assay: high-sensitivity digital droplet PCR (ddPCR) for JAK2 V617F, with recommended sensitivity threshold of <0.01%; MPL and CALR assays at sensitivity <1%;
- Sample: peripheral blood is preferred for routine monitoring (no evidence of superiority of bone marrow);
- Next-generation sequencing (NGS) post-HCT: not recommended for routine MRD surveillance given sensitivity limitations (<1% in routine settings).

MRD negativity at Day +30 most strongly predicts long-term remission; negativity at Day +100/180 still confers a survival advantage. Molecular relapse—defined as the reappearance or rising trajectory of the driver mutation allele burden—should trigger pre-emptive donor lymphocyte infusion (DLI) before morphological or clinical relapse. In the largest available combined cohort (pre-emptive DLI and/or second HCT for relapse, n = 30), complete/molecular response was achieved in approximately 39% of patients and 2-year OS was approximately 70% [68]; higher figures of 88% response and 5-year OS approaching 80% that have circulated in some secondary sources are not supported by the primary literature and have been corrected here.

## 10. Relapse Management After HCT

Relapse after allo-HCT for MF can be stratified according to EBMT definitions into molecular, cytogenetic, and morphological/clinical categories, each requiring a distinct therapeutic approach [7,11,69]. Management options include:

- Pre-emptive DLI and/or second HCT for molecular relapse: in the largest available combined cohort (n = 30), molecular/complete response was achieved in approximately 39%, with 2-year OS approximately 70%; regarded as a reasonable first-line strategy, though based on limited, retrospective, single-cohort evidence [68];
- DLI for hematological/clinical relapse: response rates and long-term survival are not well established from the available literature; the figures previously cited here were not adequately supported by the underlying sources and have been removed pending confirmation from larger, relapse-type-specific series; response to first DLI is nonetheless suggested to be an important predictor of long-term outcome;
- Hypomethylating agents (HMAs, azacitidine): restore donor chimerism in approximately 58% of cases; use remains investigational and retrospective [70];
- Second HCT for DLI-refractory relapse: outcome data derive mainly from the same limited combined cohort discussed above [68]; a single case report [71] is insufficient to support population-level response or survival estimates, and these figures should be treated as preliminary pending larger series.

EBMT retrospective data suggest that DLI alone is associated with superior median OS (76 months) compared with DLI followed by second HCT (54 months) or second HCT alone (27 months), reflecting the importance of pre-emptive intervention before full morphological relapse [8]. Prophylactic DLI and maintenance JAK inhibitor therapy post-HCT are not currently supported by prospective evidence.

## 11. Future Directions and Unresolved Questions

Despite substantial progress, several critical questions remain to be addressed through prospective investigation:

- Optimal conditioning intensity for fit older patients: RIC versus MAC in patients aged 60–70 years remains undefined by randomized data;
- Optimal treosulfan dose and platform (TTF vs. TBF vs. FluTreo): dose–response relationships and interaction with donor type require prospective validation;
- Peri-transplant ruxolitinib and novel JAK inhibitors: optimal duration, tapering schedule, and safety of continuation into conditioning require formal study;
- Post-transplant maintenance therapy: the role of JAK inhibitors, azacitidine, or other agents in high-risk MRD-positive patients after HCT;
- MRD-guided pre-emptive strategies: prospective trials testing ddPCR-triggered DLI versus watchful waiting are needed to validate the retrospective evidence base
- HCT in blast phase MF: the role of induction chemotherapy, transplant timing, and conditioning intensity in MF transforming to acute leukemia;
- Patient-reported outcomes and quality of life: systematic evaluation of functional recovery, symptom burden, and survivorship in an aging transplant population.

The establishment of a prospective randomized platform specifically in MF would address multiple of these questions simultaneously and should be a priority for collaborative transplant networks including GITMO, EBMT, and CIBMTR.

## 12. Conclusions

Allogeneic HCT remains the sole curative option for myelofibrosis and is being increasingly utilized in older and higher-risk patients. Italian GITMO data document a 163% increase in overall MPN (not MF-specific) transplant volumes from 2015 to 2025, with the over-60 age group now constituting the largest recipient cohort. Three-year overall survival rates of 59–67%, depending on donor type, represent a significant clinical advance. Improved patient selection through molecular risk stratification, pre-transplant optimization with JAK inhibitors, expanded donor platforms, and refined conditioning strategies are plausible contributors to this advance, although the manuscript’s own account of comparable RIC/MAC outcomes and the absence of randomized peri-transplant JAK-inhibitor data means this attribution should be regarded as a reasonable hypothesis rather than an established causal claim.

Emerging evidence for treosulfan-based and dual-alkylator conditioning, particularly the TTF platform, suggests a promising, but still investigational, strategy for achieving full-donor chimerism with acceptable toxicity across diverse patient populations; prospective validation against FluBu2 and TBF is still required. Dose optimization, specifically avoiding treosulfan doses of 42 g/m^2^ or higher, is associated with increased overall mortality; an effect on non-relapse mortality specifically has not been statistically confirmed. Post-transplant molecular MRD monitoring using ddPCR facilitates pre-emptive donor lymphocyte infusion at molecular relapse, where response rates appear higher than those achieved with intervention at hematological relapse, based on retrospective single/combined-cohort data.

Prospective randomized trials remain the highest priority to definitively establish optimal conditioning platforms, peri-transplant JAK inhibitor use, and MRD-guided management strategies in myelofibrosis.

## Figures and Tables

**Table 1:** Number of allogeneic hematopoietic cell transplants (HCTs) performed in Italy by disease indication, 2015–2025 (GITMO Registry, export 13 April 2026).

	2015	2016	2017	2018	2019	2020	2021	2022	2023	2024	2025
AML	697	682	815	742	791	836	805	876	879	957	938
ALL	306	330	375	335	345	350	342	341	331	313	309
NHL/HD	222	225	231	243	233	200	201	171	171	200	199
MDS	140	155	148	159	171	140	162	181	212	183	209
MPN *	65	84	71	97	110	114	107	90	129	132	171
MDS/MPN	18	24	21	37	41	38	40	57	52	57	66
CML	30	23	32	27	24	26	33	33	26	31	35
CLL	29	22	18	18	18	21	36	25	26	30	25
PCN	50	64	48	55	47	39	26	22	15	14	28

* MPN includes myelofibrosis, polycythaemia vera, and essential thrombocythaemia. MPN row highlighted in bold.

**Table 2:** Patient selection criteria for allogeneic HCT in myelofibrosis.

Age	<70 Years; Individualized Assessment Above 70 with Good PS, HLA-Matched Donor, Acceptable Comorbidities (3-yr OS ~55%)
Performance status	ECOG 0–2; Karnofsky PS ≥ 70% generally required; incorporated in MTSS score
Disease risk—PMF	DIPSS Int-2/High: immediate HCT recommended DIPSS Int-1: evaluate MTSS; benefit at ~21 months DIPSS Low: non-transplant approach preferred; benefit only after 29–45 months
Disease risk—SMF	MYSEC-PM Int-2/High: proceed to transplant MYSEC-PM Int-1: consider MTSS; individualized decision
Molecular genetics	HMR mutations (ASXL1, EZH2, IDH1/2, SRSF2, U2AF1Q157): favour HCT even at lower clinical risk TP53 multihit: consider HCT regardless of DIPSS score
JAK inhibitor response	RR6 model at 6 months: poor responders (insufficient spleen reduction + transfusion dependence) should be referred for HCT evaluation
Spleen	Massive splenomegaly must be controlled pre-HCT (ruxolitinib ≥ 3 months, splenic irradiation 3–4 Gy, or rarely splenectomy) to prevent graft failure
Comorbidities	HCT-CI score incorporated; RIC preferred for HCT-CI ≥ 3 or age > 60

**Table 3:** Donor selection hierarchy in allogeneic HCT for myelofibrosis.

HLA-Matched Sibling (MSD)	Gold Standard; Superior OS in First 3 Months Post-HCT; Early NRM Lower; Preferred if Donor Age < 60	First Choice
Matched Unrelated Donor (MUD 10/10)	Comparable long-term outcomes to MSD; preferred over older sibling (>60 yrs)	First choice if no young MSD
Haploidentical + PTCy	3-yr OS ~56–72% across cohorts (larger series ~58%; smaller series up to ~72%); CIBMTR use increased from 3% (2013) to 19% (2019); valid alternative	Second line
7/8 HLA-Mismatched Unrelated	Comparable outcomes to haploidentical; acceptable alternative	Second line
Cord Blood	Not recommended due to high graft failure risk in MF	Not recommended

**Table 4:** Conditioning regimens for allogeneic HCT in myelofibrosis: key evidence and clinical considerations.

Fludarabine + Busulfan (FluBu2—RIC)	RIC	Older/Comorbid Patients; Widely Used; 5-yr OS ~60–67%; NRM ~16–24%	Preferred RIC Backbone; PK Monitoring Recommended
Fludarabine + Busulfan (FluBu4—MAC)	MAC	Fit patients; superior RFS vs. RIC; higher NRM; 3-yr OS 51–59%	Preferred MAC for fit/younger patients
Thiotepa + Busulfan + Fludarabine (TBF)	MAC/RIC	Europe; dual alkylation increases full-donor chimerism (87–93% vs. 45% at Day 30); 5-yr OS ~71% in the TBF arm vs. 43% with single-alkylator conditioning (*p* = 0.002) [53]; other cohorts report lower 3-yr OS of 69% (single-centre, n = 37) [60] and 55% (EBMT registry, n = 187) [61], reflecting differences in patient selection, era, and study design; reduced relapse	Used at GITMO centres; strong data for chimerism
Treosulfan + Fludarabine (TREO/FLU ≤ 42 g/m^2^)	RIC	Outcomes comparable to RIC busulfan in retrospective series (not formally tested for non-inferiority); 4-yr OS 69%; engraftment 94–100%; doses ≥ 42 g/m^2^ associated with increased overall mortality but not with significantly higher NRM	Growing evidence; preferred in elderly/less fit
Treosulfan + Thiotepa + Fludarabine (TTF)	MAC/RIC	Italian multicenter retrospective analysis, n = 37 [56]: 1-yr OS 62.2%; PFS 59.6%; NRM 33.5%; engraftment 94.6%; GVHD aGVHD II–IV 21.6%; doses ≥ 42 g/m^2^ (as per the German registry cohort) associated with increased overall mortality; effect on NRM specifically not statistically significant	Promising double-alkylator; no randomised trial yet in MF
Treosulfan + Fludarabine + ATG/thymoglobulin [62]	RIC	Second HCT/salvage setting; outcome data preliminary (single-cohort experience; 3-yr OS and 5-yr DFS figures require confirmation against the primary source and are not reported as such in [62])	Salvage/second transplant setting

**Table 5:** Contemporary post-transplant outcomes in myelofibrosis by donor type. Figures are drawn from separate cohorts/registries and are not derived from a single adjusted comparison; they should not be read as a head-to-head ranking of donor types (see text for an adjusted comparison reporting HR 1.42 for haploidentical vs. matched sibling donor OS). Graft source (bone marrow vs. peripheral blood) is not stratified in this table.

3-yr OS—Matched Related Donor	Myelofibrosis: 67.4% (62.9–72.3%) Other MPN: 59.3% (53.8–65.4%)	CIBMTR 2025 (n = 740) [16]
3-yr OS—Matched Unrelated Donor	Myelofibrosis: 59.1% (56.0–62.5%) Other MPN: 53.0% (49.4–56.7%)	CIBMTR 2025 (n = 1877) [16]
3-yr OS—Haploidentical + PTCy	Myelofibrosis: 58.7% (53.0–65.0%) Other MPN: 55.7% (49.9–62.2%)	CIBMTR 2025 (n = 566) [16]
5-yr OS (large centres)	~60%	Gagelmann & Kröger, AJH 2025 [8]
1-yr relapse incidence	10–20%	Gagelmann & Kröger, AJH 2025 [8]
Non-Relapse Mortality (5-yr)	20–30%	Gagelmann & Kröger, AJH 2025 [8]
Patients aged >65 yrs	>1/3 of transplant recipients	Gagelmann & Kröger, AJH 2025 [8]
3-yr OS trend—US (2017–2022)	62.1% vs. 55.8% (2012–16) vs. 50.2% (2007–11) vs. 45.3% (2002–06); *p* < 0.0001	CIBMTR 2025 (n = 144,996) [16]

## Data Availability

No new data were created or analyzed in this study.
